# An Efficient COVID-19 Prediction Model Validated with the Cases of China, Italy and Spain: Total or Partial Lockdowns?

**DOI:** 10.3390/jcm9051547

**Published:** 2020-05-20

**Authors:** Samuel Sanchez-Caballero, Miguel A. Selles, Miguel A. Peydro, Elena Perez-Bernabeu

**Affiliations:** 1 Institute of Manufacturing and Design, Universitat Politècnica de València, Plaza Ferrandiz i Carbonell, 2, 03801 Alcoy, Spain; 2 Technological Institute of Materials, Universitat Politècnica de València, Plaza Ferrandiz i Carbonell, 2, 03801 Alcoy, Spain; maselles@dimm.upv.es (M.A.S.); mpeydro@upv.es (M.A.P.); 3 Department of Applied Statistics, Operations Research and Quality, Universitat Politècnica de València, Plaza Ferrandiz i Carbonell, 2, 03801 Alcoy, Spain; elenapb@eio.upv.es

**Keywords:** coronavirus, COVID-19, prediction, forecast, model, China, Spain, Italy, UK, France, Germany, SARS-CoV-2, Verhulst

## Abstract

The present work develops an accurate prediction model of the COVID-19 pandemic, capable not only of fitting data with a high regression coefficient but also to predict the overall infections and the infection peak day as well. The model is based on the Verhulst equation, which has been used to fit the data of the COVID-19 spread in China, Italy, and Spain. This model has been used to predict both the infection peak day, and the total infected people in Italy and Spain. With this prediction model, the overall infections, the infection peak, and date can accurately be predicted one week before they occur. According to the study, the infection peak took place on 23 March in Italy, and on 29 March in Spain. Moreover, the influence of the total and partial lockdowns has been studied, without finding any meaningful difference in the disease spread. However, the infected population, and the rate of new infections at the start of the lockdown, seem to play an important role in the infection spread. The developed model is not only an important tool to predict the disease spread, but also gives some significant clues about the main factors that affect to the COVID-19 spread, and quantifies the effects of partial and total lockdowns as well.

## 1. Introduction

A pandemic like the one caused by the COVID-19 virus is not new to humanity. Unfortunately, it is a recurring theme in history. Previous pandemics like the “Black Death” (1347–1351) or the “Spanish Flu” (1918–1919) caused many deaths and devastation in cities and countries [1]. Even these lasted more than a year, an aspect that seems impossible for the COVID-19 pandemic due to the strong measures already taken around the world. However, although it seems that we are facing another important pandemic, the amount of information generated since the first case was detected makes it special. There is no doubt that the large amount of daily data has helped in the creation of an accurate evolution model that helps governments in decision making.

In the absence of a definitive vaccine to stop the progress of this virus, each country fights against the COVID-19 expansion with a series of decisions that, of course, are never to the liking of the citizens. In addition, the economies are paralyzed, causing billions in losses.

China, where the pandemic started [2], and Italy and Spain in Europe, are the countries with the highest mortality rate so far. They have high values also in terms of the total number of infected people. It is important for the rest of the countries to know if the measures adopted by these countries are the most adequate to stop the virus expansion in the shortest time possible, thus avoiding many deaths.

The objective of this paper was to analyze the effects of the different strategies adopted by China, Italy, and Spain in order to stop the spread of the COVID-19 and determine what actions have been critical in its development. Although some authors [3,4,5,6,7] have developed other models to explain the COVID-19 dynamics, these only focus on the case of China or Japan [8]. The present work develops a model that not only analyzes China, Italy, and Spain but also is used to conduct an analysis of the partial and total lockdown strategies.

## 2. Chronology

**Figure 1:**
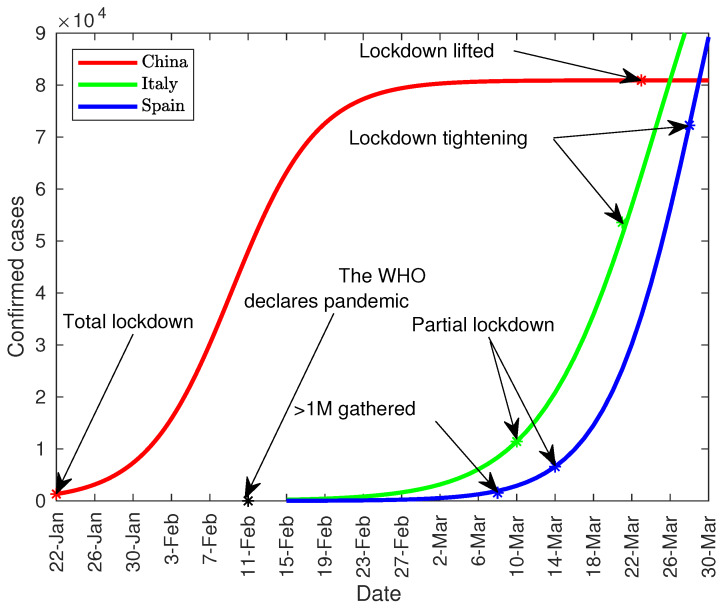
Timeline of the most relevant milestones.

The objective of this chronology is to contextualize some milestones and reflect some specific events that have occurred in Spain as well. The number of infected people shown in this chronology has been obtained from the Johns Hopkins [9] database, which in turn extracts them from the WHO among others. Table 1 shows some of milestones commented in this paper, while Figure 1 contextualizes the most relevant ones.

## 3. Development of the Predictive Model

Based on data published by the WHO and summarized by the Johns Hopkins University [9], a predictive model based on the Verhulst [28] equation has been applied:

(1) $$P\left(t\right)=\frac{KP_{0}e^{rt}}{K+P_{0}\left(e^{rt}-1\right)}$$

where P(t) represents the number of infected as a function of time, $P_{0}$ the initial infected population, *K*, the total number of infected people and *r* the growth rate of the number of infected people.

The data, Matlab code, and graphs shown in this work are presented in the Supplementary Materials.

### 3.1. Application of the Predictive Model to the Spread of the Virus in China

**Figure 2:**
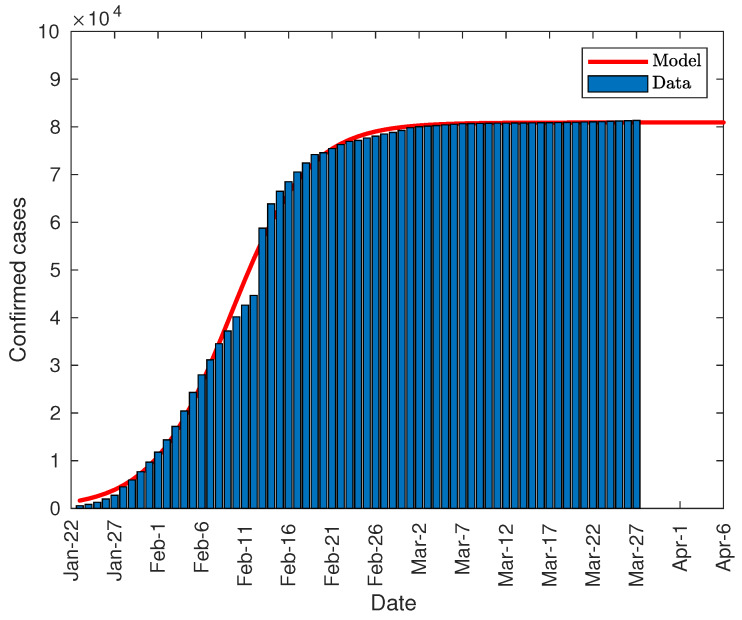
Total number of infected people in China.

The data provided by the WHO have been fitted to Equation (1) using the Matlab “Curve Fitting” tool. Figure 2 shows the evolution in the total number of infected people in China from the day after the total lockdown of Wuhan [10]. As can be seen, the model fits quite well except in the zone of February 11, date on which China changes the counting methodology of the infected people due to a lack of tests [29]. From that day, the doctors themselves made the diagnosis based on the symptoms. Despite this flaw, the model fits reasonably well with the evolution of the pandemic.

The fitting parameters used in the model have been:

*K* = 8.091 × 10^4^ (8.031 × 10^4^, 8.125 × 10^4^)
*P*_0_ = 1320 (1071, 1569)
*r* = 0.2239 (0.2135, 0.2343)

where the values in parentheses reflect the confidence intervals at 95% of such parameters. The regression coefficient is $R^{2}=0.9969$.

After fitting the data to the described function, the first derivative of the function was obtained in order to see the possibility of predicting the peak of infection using this method.

**Figure 3:**
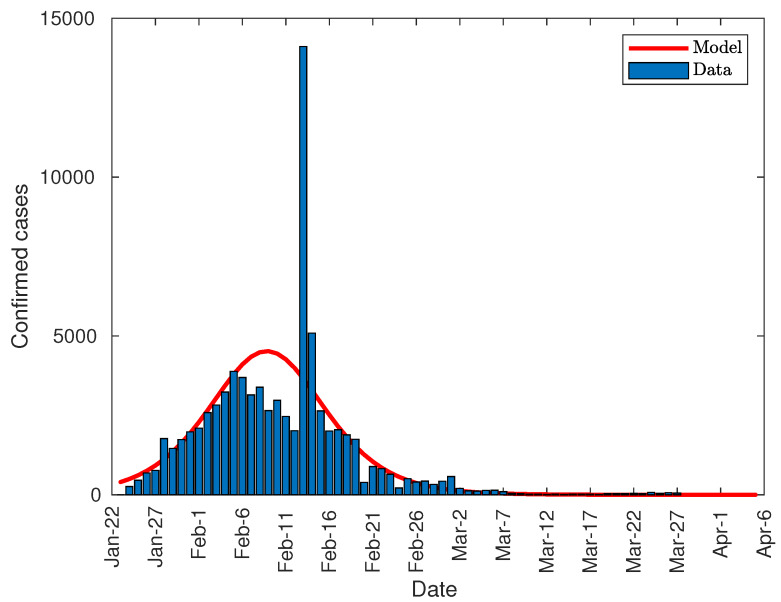
Number of daily diagnosed cases in China.

Figure 3 shows the comparison between the number of daily registered infections and the value predicted by the model. As shown, the empty area below the model curve corresponds to the peak surface registered on 14 February due to the change in methodology mentioned earlier. Therefore, the peak of infections was reached around 10 February, that is, 19 days from the start of total lockdown on 21 January; which is compatible with the upper end of the range of the incubation period established in 15.6 days for for 97.5% of infected people in Wuhan [30], up to 3 more days of virus duration on different surfaces [31].

**Figure 4:**
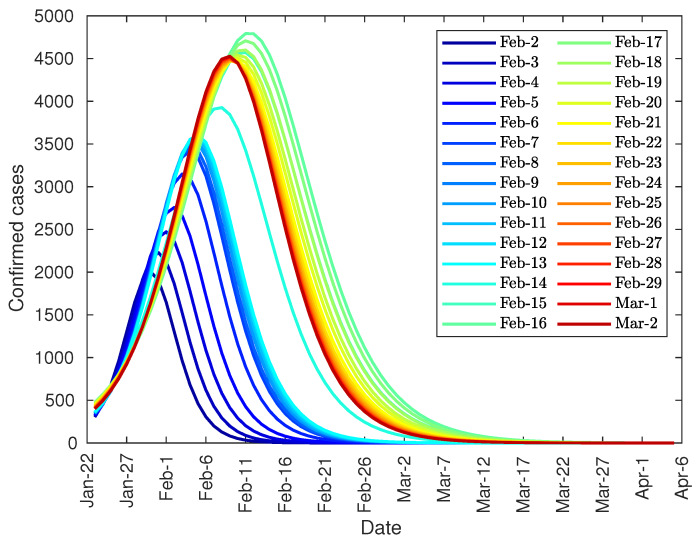
Evolution of the peak of diagnosed COVID-19 patients in China.

The arrival at that point can be predicted in advance by studying the evolution of the peak based on data from previous days. Figure 4, shows the prediction of the number of daily diagnosed cases with the data from 22 January to 2 February, and so on. As can be seen, there is a steady increase in both the height of the peak and the date until the curve stabilizes at around 4500 on 11 February. However, both the peak day and its value could not be approximated with certainty until 13 February due to the change in the method of counting coronavirus patients.

**Figure 5:**
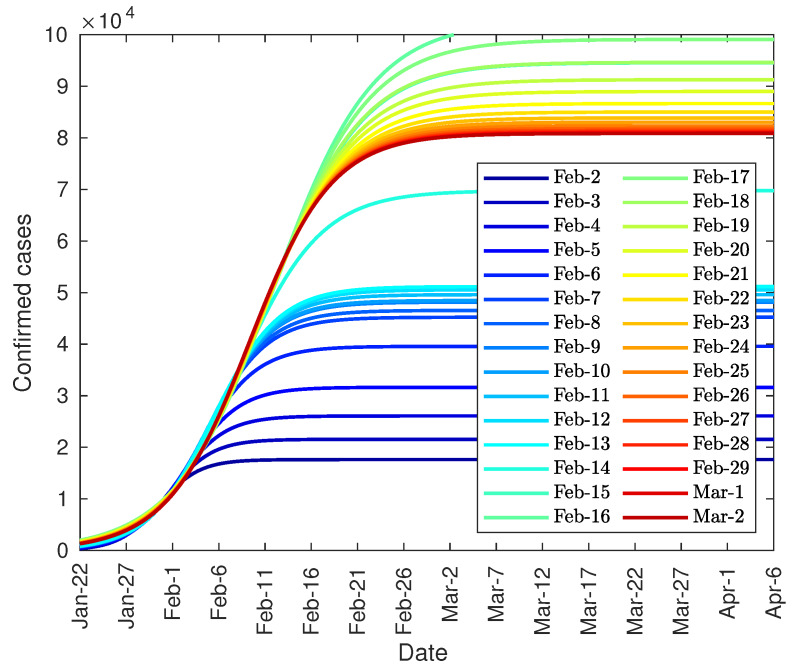
Evolution of the total number of diagnosed COVID-19 patients in China.

On the other hand, Figure 5 shows the evolution of the number of confirmed cases predicted from the data from 22 January to the indicated day. As shown, the trend is increasing initially, until 15 February, then it reaches a maximum value, due to the peak of unreported cases, and then it decreases to a value close to 80,000 diagnosed cases.

### 3.2. Application of the Predictive Model to the Spread of the Virus in Italy

**Figure 6:**
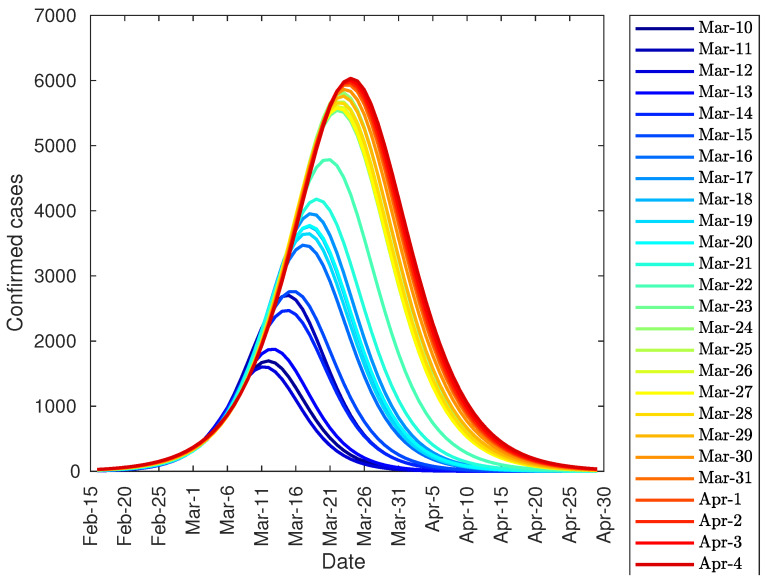
Evolution of the peak of diagnosed COVID-19 patients in Italy.

In order to predict the peak of infections in Italy, the authors have fitted the number of infections produced from 15 February onwards to Equation (1). Subsequently, the authors computed the first derivative of both the function and the data. Figure 6 shows this evolution and, as shown, the peak stabilizes around 23 March, 13 days after partial lockdown, and one day after total lockdown (with 5900 diagnosed cases) by shutting down all non-necessary businesses and industries.

**Figure 7:**
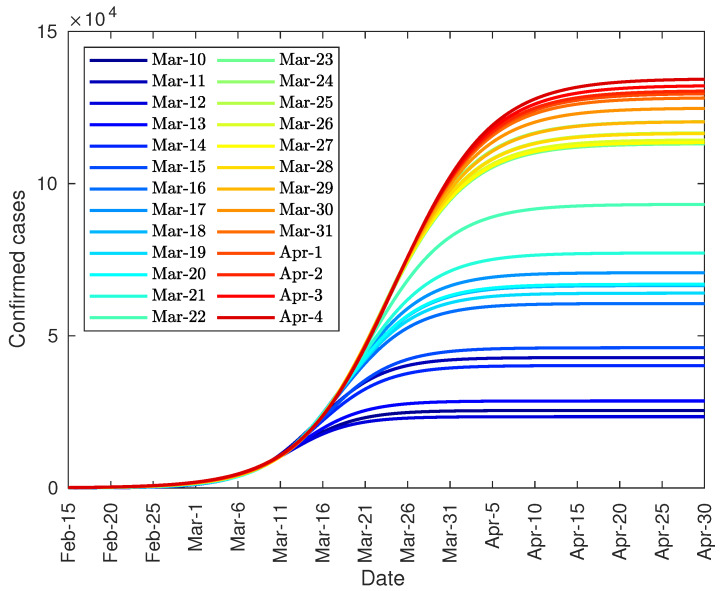
Evolution of the total number of diagnosed COVID-19 patients in Italy.

Regarding the number of diagnosed cases, Figure 7 shows the evolution of the number of diagnosed cases, which seems to stabilize over time with 130,000 cases.

**Figure 8:**
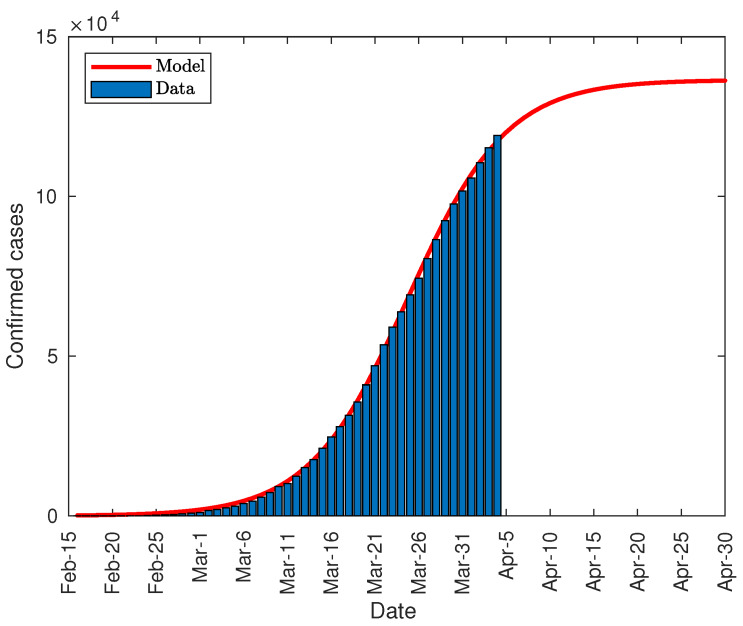
Total number of COVID-19 infections in Italy.

Figure 8 shows the prediction made on April 1, with a total number of diagnosed cases close to 136,000, standing at a range between 134,000 and 139,000 with a reliability of 95%. The fitting parameters used in the model have been:

*K* = 1.365 × 10^5^ (1.34 × 10^5^, 1.389 × 10^5^)
*P*_0_ = 139.8 (121, 158.7)
*r* = 0.1775 (0.1735, 0.1816)

where the values in parentheses reflect the confidence intervals at 95% of such parameters. The regression coefficient is $R^{2}=0.9996$. The growth rate is quite similar to that produced in the case of China.

**Figure 9:**
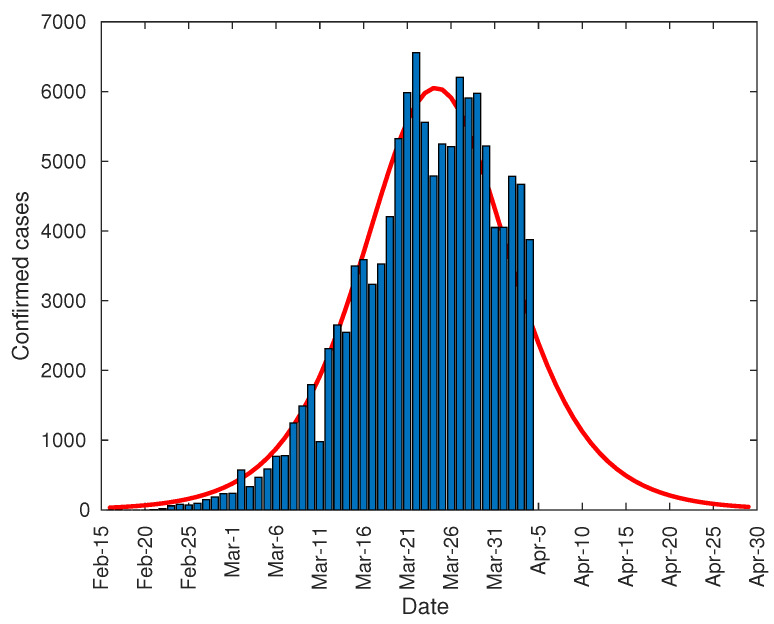
Number of daily diagnosed cases in Italy.

From this model, and computing the first derivative again, the distribution of daily diagnosed cases has been obtained, and is shown in Figure 9. As in the case of China, the data outside the prediction curve fill the gaps in the beneath area.

### 3.3. Application of the Predictive Model to the Spread of the Virus in Spain

**Figure 10:**
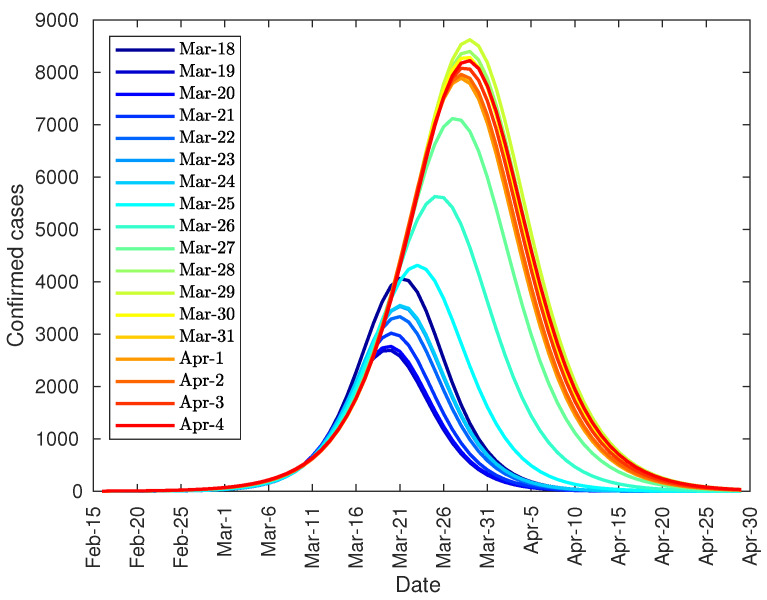
Evolution of the peak of diagnosed COVID-19 patients in Spain.

Following the same criteria as in the case of Italy, the authors have predicted the peak of diagnosed cases in Spain. Figure 10 shows the evolution of the first derivative of the fitting equation, taking the data from 15 February onwards. As shown, the peak of diagnosed cases was reached on 29 March, 15 days after the announcement of the partial lockdown (13 days after it became effective), with a prediction of about 8600 new cases per day.

**Figure 11:**
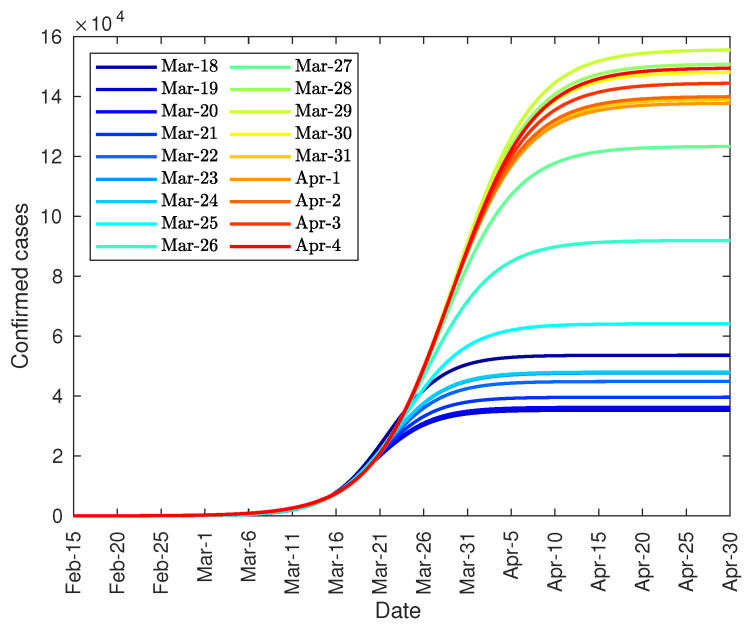
Evolution of the total number of diagnosed COVID-19 patients in Spain.

Regarding the diagnosed cases, Figure 11 shows the evolution of the number of diagnosed cases. Similarly, this number seems to stabilize over time at around 154,000 infected people.

**Figure 12:**
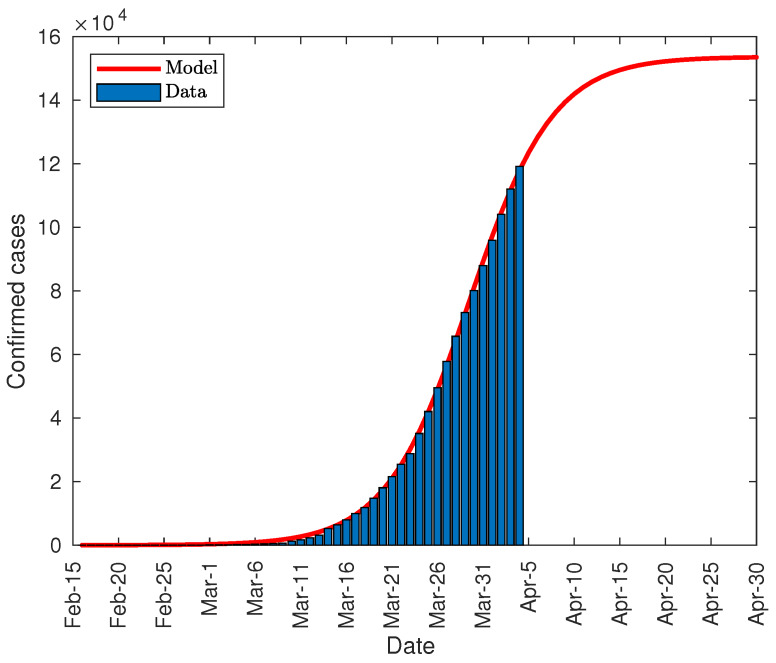
Total number of COVID-19 infections in Spain.

Figure 12 shows the prediction made on April 4, with a total of cases close to 154,000 people, ranging between 149,000 and 158,000 with a reliability of 95%. The fitting parameters used in the model have been:

*K* = 1.537 × 10^5^ (1.494 × 10^5^, 1.579 × 10^5^)
*P*_0_ = 12.21 (9.671, 14.76)
*r* = 0.217 (0.2115, 0.2226)

where the values in parentheses reflect the confidence intervals at 95% of such parameters. The regression coefficient is $R^{2}=0.9996$. The growth rate is practically identical to that of China. On the other hand, it can be seen that the initial population $P_{0}$ of the model is significantly lower than in the Italian case, which is consistent with the development of the disease in both countries.

**Figure 13:**
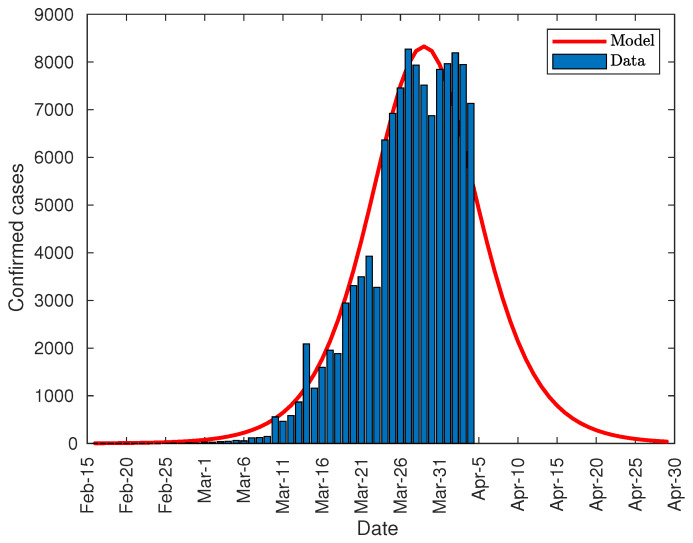
Number of daily diagnosed cases in Spain.

From this model, and computing the first derivative again, the distribution of daily diagnosed cases has been obtained, and is shown in Figure 13. As in the case of Italy and China, the data outside the prediction curve fill the gaps in the beneath area.

### 3.4. Predictive Model Parameter Identification

The model has the limitation that requires the parameter *K* (total number of infected people), which is obviously unknown. However, as can be seen in the different figures of the total number of infected, once the lower inflection point of the curve has been exceeded, this appears to become a straight line. Calculating the equation of its slope, it is possible to obtain the parameters $K,r,P_{0}$. For this purpose, the equation of the line that passes through the peak of infected cases has been calculated, and it is in the middle of the line because it is a symmetric function. The number of infected cases at this point is K/2, and the time required to reach that point can be calculated with the condition P(t)=K/2. Solving the equation, the time *t* required to reach that value can be calculated as follows:

(2) $$t=\frac{\log(\frac{K-P_{0}}{P_{0}})}{r}$$

Once the time required to reach the peak of infections is calculated, we have done a series expansion centered on that point, obtaining the following equation:

(3) $$P_{series}\left(t\right)=\frac{K}{2}\left(1-\frac{1}{2}\log\left(\frac{K-P_{0}}{P_{0}}\right)\right)+\frac{1}{4}Krt=a+bt$$

where *a* is the ordinate at the origin and *b* is the slope. In this way, after exceeding the inflection point and knowing the number of infections ($P_{1}$) on day ($t_{1}$) and ($P_{2}$) on day ($t_{2}$), it is possible to obtain the parameters *a* and *b*. Starting from these parameters, and adding between 13 and 19 days from the first day of lockdown, the peak of infected people is achieved, that is, K/2. Analytically expressed, it would be as follows:

(4) $$K=2(a+b\left(t_{lockdown}+t_{incubation}\right))$$

The rest of parameters can be easily computed from a,b,K. Table 2 shows a comparison of the prediction made just after the inflection point in the three countries. The number of days is counted from the start of the series, which is 22 January in the case of China and 15 February in the cases of Italy and Spain. As shown, the values of the total cases are very adjusted despite using a relatively low number of diagnosed cases.

### 3.5. Predictive Model Forecasts

**Figure 14:**
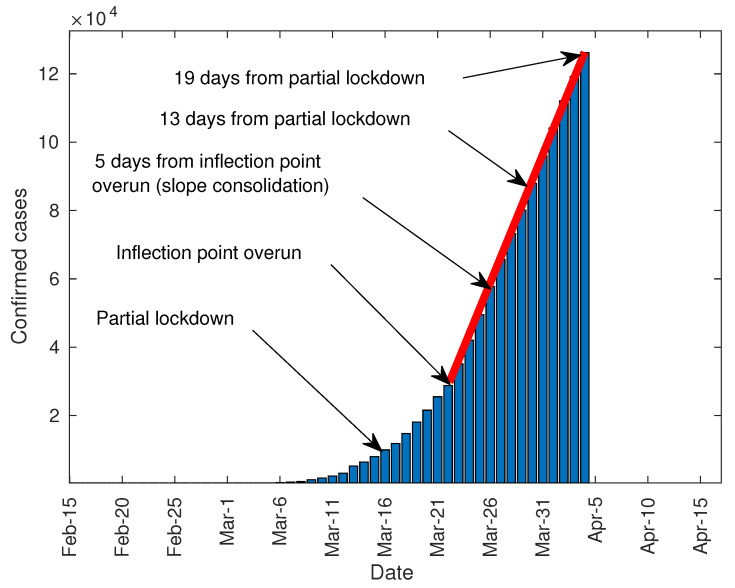
Slope determination for Spain.

While the predictive model in Section 3.1, Section 3.2 and Section 3.3 allows predicting the outreach of the infection peak, the predictive model in Equation (4) allows forecasting the number of infected people considering the number of days from the lockdown to the infection peak. On 4 April, it is difficult to ensure that the infection peak has been outreached in Italy and Spain, as the number of daily diagnosed cases does not show a clear downward trend. Moreover, considering the precedents in China where the infection peak took place 19 days after the lockdown, it is reasonable to think that the peak has not been outreached in Italy and Spain. Furthermore, it seems contradictory that the draconian lockdown imposed in China requires more time to take effect. Thus, it is logical to consider a scenario where the infection peak is outreached in 19 days or even more in both countries. With this aim, two scenarios were considered. The first one supposed that the peak is surpassed after 13 days, while the second considered this to happen after 19 days. To forecast both scenarios, the slope of the curve of total infected people was computed from the straight part, as shown in Figure 14.

**Figure 15:**
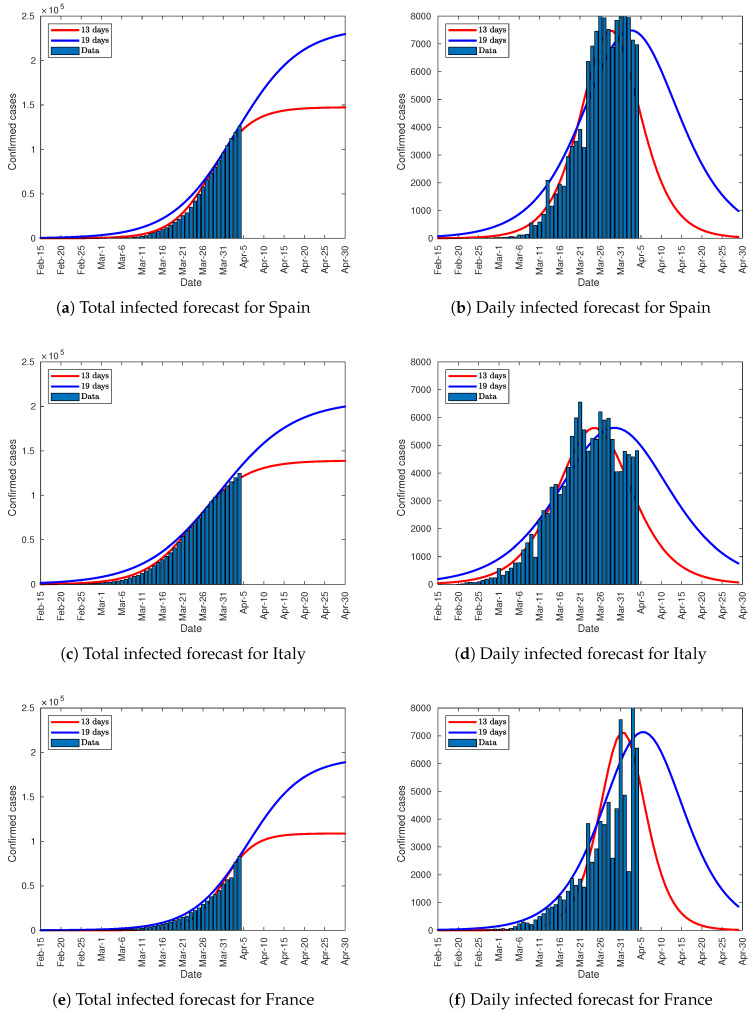
Total and daily infected forecasts.

**Figure 16:**
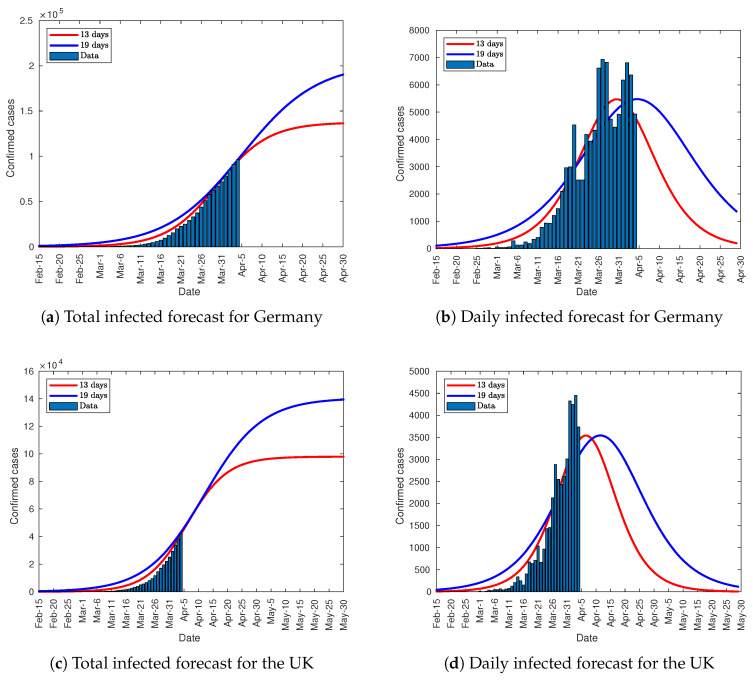
Total and daily infected forecasts.

The rest of parameters of the Verhulst equation were computed trough Equation (4). Table 3, Figure 15 and Figure 16 show the results and the forecasts as well. Besides the forecast for Italy and Spain, France, Germany, and the UK have also been analyzed with this method. As these countries are far away from their infection peak on April 4, the method shown in Section 3.1, Section 3.2 and Section 3.3 cannot predict the total number of infected people. The parameter $K_{13}$ in Table 3 represents the total infected people in a scenario where the infection peak is outreached in 13 days from the lockdown for each country. On the other hand, $K_{19}$ describes a scenario where the peak is outreached in 19 days. The curves in Figure 15, and Figure 16 depict both scenarios. As can be seen, the forecast for Italy and Spain is quite similar to the one in Section 3.2 and Section 3.3. Conversely, the predictions for the rest of the countries, and especially for the UK, must be embraced with caution as the growth slope is not fully consolidated. To get a reliable forecast, 5 days of data after clearly surpassing the lower inflection point is needed, as shown in Figure 14. The number of infected people from 13 to 19 days after lockdown is the half of the overall number.

## 4. Results and Discussion

**Figure 17:**
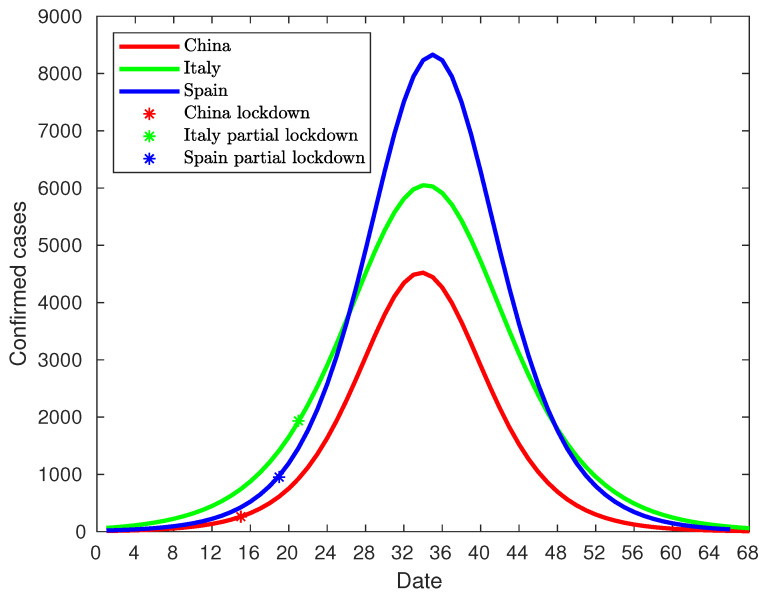
Number of daily diagnosed cases comparison.

**Figure 18:**
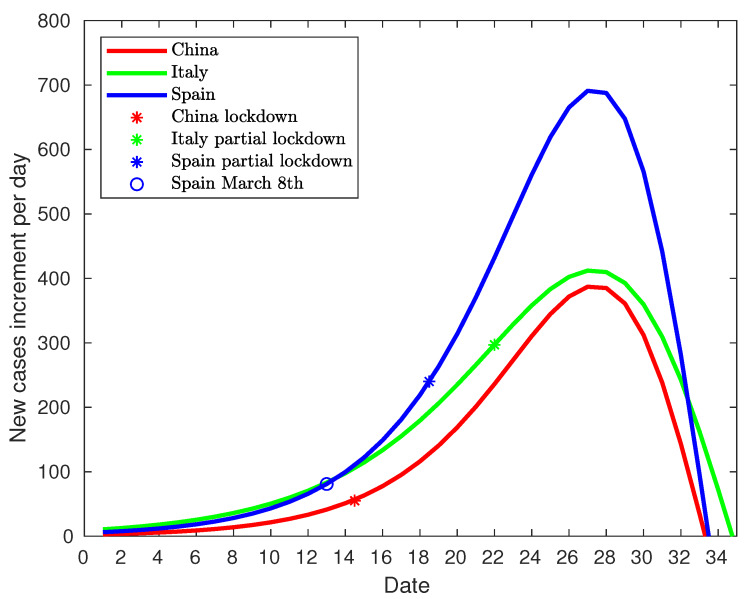
Comparison of centered rate of new infections.

After the development of the model for the three countries, it is possible to make a comparison between them. Since the spread of the epidemic takes place at different time intervals, the models have been shifted to coincidence at the peak of infections. Figure 17 shows this comparison. The distribution of infected cases has a similar duration, around 68 days, and it can be stated that the type of lockdown does not have a significant influence on the duration of the virus spread. However, there is a significant difference in the peak of infections, which may have been due to three causes. The first one is related to the lockdown type. While China established a total lockdown, Italy and Spain started with partial lockdowns. The peaks in Italy and Spain are significantly higher than the one in China but are also very different from each other, so there must be other additional causes.The second cause is the infected population at the beginning of lockdowns. China had 571 infected people when the lockdown was enforced. Italy had 12,462 and Spain 5,678 (9,942 on the day of enforcement). In this case, Italy and Spain had a number of infected people higher than in China; but Spain, with fewer infected people, had a higher peak than Italy, so other additional factors have an influence on this peak. The third cause is the rate of new infected people at the beginning of lockdowns. Figure 18 shows the comparison between the rate of new cases (acceleration of infection) for the three countries centered on the day of maximum rate. At the beginning of the lockdown in China, the growth rate amounted to 55 new daily infections, while in Italy to 297, and in Spain to 240. As shown, the rates of new cases in China and Italy show the same behavior, with a vertical offset of Italy with China. Italy and Spain initially present a very similar trend, breaking rapidly from day 12 of the graph (Day 33 corresponds to the peak of cases on 29 March (null rate), and if we take 21 days off until day 12, it corresponds to 8 March), which in the case of Spain corresponds to 8 March. On the other hand, if the graph of daily diagnosed cases is analyzed, there is a spike that abruptly surpasses the model on 13 March (see Figure 13). Given that the median of the incubation period is 5 days [30], such a spike must be due to causes that happened 5 days before, that is, March 8. Both indicators point in the direction that on that day there is a significant change in the evolution of the pandemic in Spain, explaining the difference in the evolution between Spain and Italy, even though the same measures were taken and the numbers of diagnosed cases at the beginning of partial lockdown were similar. As stated in the chronology section, a large number of massive gatherings took place that day that may have been the cause of such a spike and the subsequent change in trend.

Therefore, if Spain is removed from the analysis, the comparison between Italy and China shown in Figure 18 reveals that the rate of new infections of Italy and China follows the same pattern. The curve from Italy is similar to that of China if it is vertically shifted. As this parallelism is sustained after the lockdown in Italy, it can be concluded that the rate of new infections is not affected by the type of lockdown. Thus, if the rate of new infected is removed, the infected population size at lockdown time is the only significant parameter. In consequence, the infection peak and the total infected population are mainly due to the infected population size at the lockdown regardless of a total or partial one.

Concerning the described exodus of many Madrid citizens to other areas in Spain, produced between 12–14 March, it seems that it has not had a significant effect on the COVID-19 spread in Spain, as the daily infection scatter plot does not exhibit a spike over the model between 17–19 March. The attenuation of the effect of this event was caused undoubtedly by the declaration of the state of alarm on 14 March, which retained the displaced infected people, restraining thus the COVID-19 spread.

## 5. Conclusions

In the light of the results obtained after the analysis of the COVID-19 disease spread over the three countries, it can be concluded that the major parameter in the disease spread is the infected population size at lockdown time, regardless of the kind of lockdown applied, provided that the rate of new infections is not out of control. The delay in putting in place isolation measures can produce an infection spike that triggers the rate of new infections as it has happened in Spain. The Spanish case is a clear example of how a punctual public event caused an infection spike that increased the rate of new infections, and how the infection spread caused by the exodus of some Madrid citizens was mitigated due to the isolation measures adopted two days later. Additionally, the data show that the infection peak is reached from 12 to 19 days after lockdown, regardless of the type. Thus, the epidemic lifespan is about 68 days, and it seems to be lockdown-independent. The lockdown delay seems to slightly extend the lifespan. According to Figure 18, on the same timescale, Italy delayed the lockdown two days more than Spain, thus reaching the infection peak one day later.

As after the disease remission the appearing of a new outbreak is quite probable, there are some guidelines that we consider important, according to the previous analysis, to avoid future lockdowns. First of all, the development of a real-time database with universal access is it is highly advisable, registering the maximum quantity of data related to the infection. Such as day, city, date, sex, age, and symptomatology. This would allow the development of prediction models in a fast way, even for researches from other areas of science, as this case. Secondly, it’s also important the development of a big data analysis system capable of tracking the spike rise in the daily infection curve. The emergence of a spike can point to a critical event, as occurred on March 13 in Spain. And finally, it is also recommended to develop a fast response system to conduct the immediate isolation of all the people that could be related to a critical event for a later diagnose after such event discovery. This response must occur during the following 24 h, as this time is enough to trigger the rate of new infections. This requires, besides the mentioned guidelines, the development of a geolocation warning system capable making the affected people aware of the danger. These recommendations are in line with the measures adopted by countries like Korea [32] and Singapur [6], who have kept the infection spread to significantly low levels.

Finally, it can be noted that the developed model can be used, not only to predict the total number of infected people, but also the daily infection curve, and the infection peak. As one of the equation parameters is the total number of infected people, it is not possible to make predictions before the curve inflection point. However, after the inflection point overrun, the model provides some important tools. Firstly, it can be used to accurate predict the daily number of infected people during the following 2–3 days from actual day. Secondly, the model can predict the total number of infected people. And finally, the evolution of the daily infected curve can be used to predict the infected peak size and date, as this peak tends to converge to its final shape as shown in Figure 4, Figure 6 and Figure 10.

The model has also been applied to forecast the COVID-19 cases in Germany, France, and the UK, demonstrating that the model is reliable.

Therefore, the model is an efficient tool to predict with many days in advance the total amount of people infected, and also the daily new cases. It also gives an approximate date of the infection peak with a minimum error in each country. This will help governments to better plan their hospital and health capabilities. 

## Figures and Tables

**Table 1:** Relevant milestones.

Date	Location	Milestone
22 January	China	The Chinese government decrees the total lockdown of the area of Wuhan [10].
31 January	Spain	First COVID-19 case diagnosed [11,12].
11 February	World	The WHO declares the global pandemic [13].
12 February	Barcelona	The world’s largest mobile phone showcase, Mobile World Congress (MWC),
		is cancelled over coronavirus concerns [14].
13 February	Spain	The first coronavirus related death occurs. This would be known on 3 March
		after an autopsy [12].
8 March	Spain	All sorts of sports competitions are held at all levels. Only in the first soccer
		division, up to 760,000 people gathered throughout Spain [15]. The 8 M
		demonstrations gathered more than 550,000 people throughout Spain [16].
9 March	Spain	All primary and high schools and universities in Madrid are closed, and
		teleworking was recommended [17].
10 March	Italy	The Italian Prime Minister declares a partial lockdown, which restricts the
		mobility of Italians but allows industrial activity [18].
12 March	Spain	The closure of schools and universities is extended to the whole country.
13 March	Spain	The Spanish president announces the declaration of the state of alarm for the
		next day [19]. The exodus of people from Madrid to other towns throughout
		Spain is detected.
14 March	Spain	First day of the state of alarm [20]. This leads to partial lockdown and
		the restriction on movements, but it allows the industrial activity, in a similar
		way to Italy [21].
16 March	Germany	The Deutch Chancelor bans gatherings and closes shops, bars and churches
		while urges the citizens to stay at home [22].
17 March	France	The French Prime Minister orders people to stay at home and only go out for
		essential duties [23].
21 March	Italy	The Italian Prime Minister declares total lockdown and stops all industrial
		activity in Italy, except the essential for the country [24].
23 March	China	China lifts quarantine over Wuhan after two months of total lockdown [25].
	UK	The Prime minister of the United Kingdom declares partial lockdown. Shops
		selling non-essential goods are told to shut and gatherings in public of more
		than two people who do not live together are prohibited [26].
28 March	Spain	The president of the Spanish government announces the tightening of lock-
		down, allowing only the development of activities considered essential [27].
		The enforcement of the total lockdown will begin on Tuesday, 31 March.

**Table 2:** Parameter K identification.

	China	Italy	Spain
$t_{1}$	12	30	37
$t_{2}$	13	31	38
$P_{1}$	17,200	24,750	28,770
$P_{2}$	20,440	27,980	35,140
a	−21,680	−72,150	−206,920
b	3240	3230	6370
$t_{lockdown}$	0	25	29
$t_{lockdown}+t_{incubation}$	19	38	42
K	79,760	101,180	121,240

**Table 3:** Forecast of the total number of COVID-19 infections for Spain, Italy, France, Germany and the UK.

	Italy	Spain	France	Germany	UK
$t_{1}$	35	37	44	37	43
$t_{2}$	44	50	50	50	50
$P_{1}$	47,021	28,770	40,174	24,873	17,089
$P_{2}$	97,689	126,168	83,019	96,092	41,903
a	−150,021	−248,440	−274,023	−177,827	−135,340
b	5629.78	7492.15	7140.83	5478.38	3544.86
$t_{lockdown}$	25	29	32	32	38
$K_{13}$	139,081	147,445	108,910	137,400	97,985
$r_{13}$	0.1619	0.2032	0.2623	0.1595	0.1447
$P_{0_{13}}$	251.228	23.60	0.6276	104.897	52.833
$K_{19}$	206,638	237,351	194,600	203.141	140,524
$r_{19}$	0.1090	0.1263	0.1468	0.1079	0.1009
$P_{0_{19}}$	1521.26	487.05	94.26	825.53	402.551

## Supplementary Materials

The data, matlab code and graphs are available online at Github: https://github.com/sasanca/COVID-19Model. The forecast for Spain and Italy will be daily updated until the end of the pandemic.
